# Do vitamin D and high-sensitivity-C reactive protein levels differ in patients with hyperemesis gravidarum? A preliminary study

**DOI:** 10.4274/tjod.76753

**Published:** 2016-09-15

**Authors:** Saynur Yılmaz, Derya Akdağ Cırık, Canan Demirtaş, Hakan Timur, Ayşe Şahin, Nuri Danışman, Dilek Uygur

**Affiliations:** 1 Etlik Zübeyde Hanım Women’s Health Training and Research Hospital, Clinic of Obstetrics and Gynecology, Ankara, Turkey; 2 Gazi University Faculty of Medicine, Department of Biochemistry, Ankara, Turkey; 3 Zekai Tahir Burak Women’s Health Training and Research Hospital, Clinic of Obstetrics and Gynecology, Ankara, Turkey

**Keywords:** C-reactive protein, hyperemesis gravidarum, inflammation, Vitamin D

## Abstract

**Objectives:**

The high sensitivity-C reactive protein (hs-CRP) is an inflammatory marker and vitamin D is an immune modulator that might play a critical role in the pathogenesis of hyperemesis gravidarum. Therefore, in the current study, we tested the hypothesis that suggests women with hyperemesis gravidarum have lower 25-hydroxyvitamin D levels and higher hs-CRP levels, compared to controls.

**Materials and Methods:**

This prospective case-control study included 30 women with hyperemesis gravidarum (study group) and 30 age- and body mass index-matched healthy women (control group). The levels of 25-hydroxyvitamin D and hs-CRP were compared between two groups.

**Results:**

Both the serum 25-hydroxyvitamin D (5.30 μg/L vs. 6.44 μg/L; p=0.09) and hs-CRP levels (0.29 mg/dL vs. 0.47 mg/dL; p=0.93) were not significantly different between the study and control groups. Vitamin D deficiency was present in 27 (90.0%) women in the study group and 22 (73.3%) women in the control group (p=0.181). There was also no correlation between 25-hydroxyvitamin D and hs-CRP levels in both groups.

**Conclusion:**

Although it did not reach statistical significance, vitamin D levels were lower in the study group compared with controls. Therefore, vitamin D might be speculated to play a crucial role in controlling the inflammatory status associated with hyperemesis gravidarum. Larger studies are required to clarify whether there is a relation between vitamin D deficiency and hyperemesis gravidarum.

## INTRODUCTION

Hyperemesis gravidarum (HG) is a common medical problem that affects nearly 1 percent of pregnant women, and causes morbidity both for the mother and fetus^(1)^. The etiology is still unclear and until now, many theories have been introduced. Psychological factors, hormonal changes, gastrointestinal dysmotility, and immunologic dysregulation have been proposed as possible causes^(2,3)^. In recent years, a close relationship was also recently found between HG and inflammation caused by helicobacter pylori infection^(4,5)^.

Vitamin D is a well-known immunomodulator and anti-inflammatory agent in the body. Vitamin D deficiency is shown as the one of the major causes of many diseases in the reproduction system^(6,7)^. Sugito et al.^(8)^ found that pregnant women with HG had increased cell-free DNA levels in blood, which was believed to emerge as a result of hyperactivity of the maternal immune system and destruction of trophoblasts. However, fasting, which is a feature of HG and pregnancy, are also known to weaken the human and cell-mediated immune system. Although both pregnancy and fasting are features of HG, in contrast to expectations, the immune system was activated in women with HG. It might be speculated that vitamin D has a key role in the etiopathogenesis of HG, and vitamin D deficiency might explain the immune theory of HG because vitamin D deficiency might lead to problems in immune regulation.

Therefore, in the current study, our objective was to compare the 25-hydroxyvitamin D [25 (OH) D] and C reactive protein (CRP) levels between women with HG and controls.

## MATERIALS AND METHODS

A total of 30 women hospitalized with a diagnosis of HG as a study group and another 30 pregnant women who were matched to the HG group in terms of age, body mass index (BMI), and gestation period as a control group were enrolled into this prospective case-control study. The Institutional Review Board of the hospital (24.07.2013/, number 8) approved the current study and all patients gave written informed consent. This study was performed in accordance with the Declaration of Helsinki and patients were reviewed from a tertiary referral center between January 1^st^, 2013 to March 3^rd^, 2013. The inclusion criteria were as follows; age 18-35 years, between 6 and 12 gestational weeks, singleton pregnancy with healthy fetus, persistent nausea and vomiting (more than 4 times/day), presence of ketosis (>80 mg/dL in a urine specimen), and weight loss (more than 5% weight loss since pregnancy).

Patients who had multiple pregnancy; trophoblastic disease; habitual abortus; any systemic disease such as diabetes hypertension or thyroid disease; psychiatric disorder; any inflammatory disorder such as urinary tract infection; and those who used antiemetic medication or any kind of medication that could potentially affect hormones were excluded from the study. All participants underwent sonographic examination to confirm the gestational week, fetal heart rate, and the absence of placental pathology. When the difference between sonographic measurement and last menstrual date were more than 3 days, crown rump length measurement was used. Weight and height measurement were used to calculate BMI.

### Biochemical measurement of vitamin D and high sensitivity-C reactive protein

Patients gave venous blood for biochemical tests following overnight fasting. Serum samples were centrifuged at 5000 revolutions/minute for 10 minutes within 20 minutes of blood sampling. Samples are stored at -80 °C. Total 25-OH-vitamin D levels in the plasma were measured using an ImmuChrom column (IC 3401 rp) kit with immune chromatographic methods. According to the instructions, the analytic detection limit of kit was 5.8 nmol/L and reference intervals were 10-60 μg/L in winter. High sensitivity-CRP (hs-CRP) levels were measured in serum using immunonephelometry (Cardio Phase hs-CRP, Siemens, Germany). In accordance with a statement for healthcare professionals from the American Heart Association/Centers for Disease Control and Prevention, hs-CRP levels were classified as follows: hs-CRP <1.0 mg/dL as low, hs-CRP between 1.0-3.0 mg/dL as intermediate, and hs-CRP >3.0 mg/dL as high levels^(9)^.

### Statistical Analysis

The study data were analyzed using the Statistical Package for Social Sciences (SPSS) version 15.0 for Windows (SPSS, Chicago, IL). In order to see if the variables had normal distribution, histogram and Shapiro-Wilk tests were used. The mean plus/minus standard deviation, median (minimum-maximum), count and percentile are presented in accordance with the distribution of the data. Categorical variables were analyzed using Fisher’s exact test and chi-square tests. As a statistical method, Student’s t-test was performed for normally distributed variables and the Mann-Whitney U test was used to analyze non-normally distributed variables. Spearman’s correlation test was used to test the strength of correlation between the variables. A p value less than 0.05 was considered as statistical significance.

## RESULTS

Age, gestational period, parity, and BMI were similar in the study and control groups (p>0.05) (Table 1). The was also no difference in hs-CRP and vitamin D concentrations between the study and control groups. Table 2 shows the comparison of hs-CRP levels and vitamin D levels in both groups. In the study group, 23 (76.7%) patients had low hs-CRP levels, 3 (10.0%) had intermediate, and 4 (13.3%) had high levels of hs-CRP. For the control group, 27 (90.0%) patients had low levels of CRP and 3 (10.0%) had intermediate levels of CRP; no significant difference was detected between the study and control groups regarding CRP levels (p=0.115). In the study group, 27 (90%) patients had low vitamin D levels, and 3 (10.0%) had high vitamin D levels. In control group, 22 (73.3%) patients had low levels of vitamin D, and 8 (26.7%) had high vitamin D levels; no significant differences were found between the study and control groups regarding vitamin D levels (p=0.181) (Table 2). No correlation was found between vitamin D and hs-CRP concentration in either the HG group or controls (p>0.05) (Table 3).

## DISCUSSION

Immune dysregulation and inflammation are suggested to have a critical role in the etiopathogenesis of HG^(10,11)^. hs-CRP is a well-known inflammatory marker and vitamin D is an immune modulator and anti-inflammatory that plays a crucial role in the reproductive system. Therefore, we hypothesized that pregnant women with HG should have lower 25 (OH) D levels and higher hs-CRP levels compared with controls, and tested this hypothesis in this prospective case-control study. To our knowledge, the current study is the first trial in the existing literature to investigate the association between vitamin D concentrations and HG. Although it did not reach statistical significance, vitamin D levels were lower in the HG group compared with controls (p=0.090). In addition, no difference was found in hs-CRP concentrations between the two groups.

In a review that analyzed various factors that contribute to the diagnosis of HG, only helicobacter pylori was identified as having a definitive impact in the etiopathogenesis of the disease^(4)^. Endoscopy conducted on women with HG proved that 90% had mucosal inflammation and helicobacter pylori activation in the stomach^(12)^. However, not all pregnant women with helicobacter pylori exhibit the signs of HG and these women are possibly inclined to helicobacter pylori because of the problems in humoral and cell-mediated immunity^(13)^. Leylek et al.^(10)^ supported this hypothesis by showing an increase in immunoglobulin, complement, and lymphocyte counts in patients with HG, as a result of immunologic activation. Vitamin D has a pivotal role in many diseases of the reproductive system as an immune modulator and anti-inflammatory agent. Vitamin D receptors might be found on a large number of immune cells. Vitamin D helps fetal immune adaptation by inhibiting the secretion of cytokines from T-helper cells^(14)^. In addition, it inhibits the secretion of pro-inflammatory cytokines from the placenta and suppresses the inflammatory response. Recent studies focused on the possibility that deficiency of vitamin D might be related with many maternal and fetal adverse outcomes such as spontaneous abortion, preterm labor, intrauterine growth restriction, and preeclampsia^(15,16)^. The present study emphasizes the possibility that vitamin D, which is known to have numerous roles in the reproductive system, might also have an impact on HG CRP is an acute phase reactant and its synthesis is primarily stimulated by IL-6 and tumor necrosis factor as a reaction to infection and inflammation^(17)^. Kuscu et al.^(18)^ also reported that women with HG had increased levels of IL-6 levels and successful treatment of resistant cases with steroid treatment might be explained by the fact that steroids have anti-inflammatory effects. Therefore, hs-CRP levels might be speculated to increase in women with HG. To our knowledge, there is only one published study that investigated hs-CRP concentrations in women with HG^(19)^. In that case-control study, researchers evaluated 56 women and described an increase in hs-CRP levels in women with HG. However, in the current study, no difference was detected in hs-CRP levels between the study group and controls. Many factors such as socioeconomic status, dietary intake of carbohydrates, and smoking were also related to variations in CRP concentrations^(20)^. The present study is a preliminary study and had a small number of patients; therefore, we might not have homogenized the two groups with these factors and thus failed to detect the difference in hs-CRP levels.

## CONCLUSION

In the present study, vitamin D concentrations were lower in the HG group compared with controls, albeit the relation was not statistically significant. We might not have been able to reach definite results and clearly explain the role of vitamin D in the etiopathogenesis of HG because our study had a small number of patients. However, whether vitamin D has an impact on the etiopathogenesis of hyperemesis or inflammation underlies the disease that causes vitamin D levels to drop needs to be clarified. Therefore, further studies with higher numbers of patients are required to investigate the association between vitamin D and HG.

## Figures and Tables

**Table 1:**
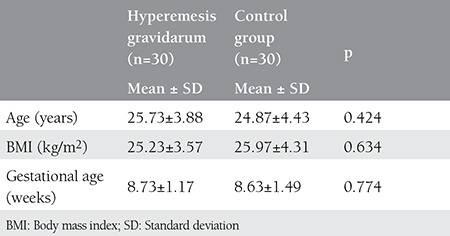
Distribution of age, body mass index, and gestational age according to the study and control groups

**Table 2:**
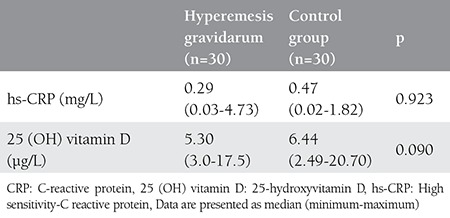
Distribution of the high sensitivity-C reactive protein and vitamin D levels in the study and control groups

**Table 3:**
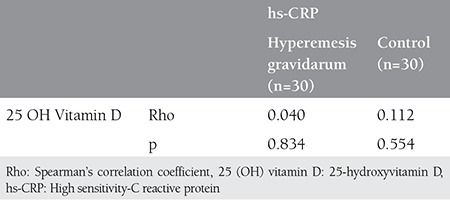
The correlation between high sensitivity-C reactive protein and vitamin D levels in the study and control groups
